# Chronic Low Back Pain: Prevalence, Impact on Quality of Life, and Predictors of Future Disability

**DOI:** 10.7759/cureus.45760

**Published:** 2023-09-22

**Authors:** Enas Alfalogy, Sahar Mahfouz, Samah Elmedany, Nahla Hariri, Salah Fallatah

**Affiliations:** 1 Family Medicine, Faculty of Medicine, Suez Canal University, Ismailia, EGY; 2 Community Medicine and Pilgrims Healthcare, Faculty of Medicine, Umm Al-Qura University, Makkah, SAU; 3 Rheumatology & Rehabilitation, Faculty of Medicine, Zagazig University, Zagazig, EGY; 4 Rheumatology & Rehabilitation, Faculty of Medicine, Tanta University, Tanta, EGY; 5 Orthopedic Surgery, Faculty of Medicine, Umm Al-Qura University, Makkah, SAU

**Keywords:** primary healthcare center, quality of life (qol), disability, prevalence rate, low back pain

## Abstract

Background: In primary healthcare settings, chronic low back pain (cLBP) is a widespread health issue with a great global impact.

Methods: A cross-sectional analytical study was performed on 918 adults attending primary healthcare settings in Makkah, Saudi Arabia, to estimate the cLBP prevalence, its effect on health-related quality of life (QOL), and predictors of subsequent disability. The visual analog scale of pain (VAS), World Health Organization Quality of Life Scale (WHO-QOL), and Oswestry low back pain disability index (ODI) were used to assess back pain severity, impact on QOL, and physical disability, respectively.

Results: The prevalence of cLBP among adults attending primary care settings in Makkah City was 25.9%, and 88.6% of them had a limited range of motion of the spine. About 58.1% had abnormal radiological findings detected by MRI. Based on the VAS scale, most patients (83.8%) had mild pain. The median (IQR) QOL score was 70 (60-80). Minimal and moderate disability scores were prevalent among 16.2% and 65.7% of patients, respectively. Disability scores were independently predicted by a lower QOL score (beta = -0.39, 95%CI = -0.49 to -0.29, p < 0.001) and higher VAS scores (beta = 0.47, 95%CI = 0.38-0.56, p < 0.001). Furthermore, disability was independently associated with having post-void residual volume (PVRV) (beta = 5.84, 95%CI = 1.97-9.72, p = 0.004) and abnormal X-ray findings (beta = 7.10, 95%CI = 1.77-12.4, p = 0.010).

Conclusion: cLBP is common among adults attending primary care settings in Makkah City; it is associated with moderate disability and lower health-related QOL.

## Introduction

Low back pain (LBP) continues to be a major health problem. Despite the significant advances in diagnostic modalities, identifying the specific cause of LBP continues to be challenging [1]. The term "low back pain" (LBP) is any discomfort that originates from the area between the 12th rib and inferior gluteal folds, regardless of whether it is associated with the referred pain [2]. About 84% of the adult population complains of LBP at some time [3]. Although 80-90% of LBP has a benign course and can be recovered within six weeks. regardless of the treatment modality [2], 5-15% develop chronicity [4]. Episodes of acute LBP, lasting up to four weeks, are usually limited for most patients, while subacute LBP is diagnosed when the pain lasts longer than four weeks, but less than 12 weeks, and the episode may last longer than 12 weeks to become chronic back pain [4,5].

Chronic LBP (cLBP) has been considered a common longstanding problem in the primary healthcare setting, with a significant burden in developed and developing countries. It may lead to long-term disability, economic loss due to decreased work hours, and frequent healthcare service use [4,5]. Low health-related quality of life (HRQOL), high functional disability, poor physical health, and high perceived pain are all indicators of significant societal costs [6]. In addition to its physical effects, cLBP has a considerable psychological impact on patients and an increased prevalence of depression, anxiety, and sleep disturbances compared with healthy people [7,8]. cLBP is also concomitant with the overuse of healthcare services and significant economic and work loss [9]. Sedentary lifestyles and high socioeconomic levels are the main contributors to the increased prevalence of LBP in recent years [10]. Therefore, LBP is considered a major health problem with a significant burden due to its clinical, economic, and social importance, which affects communities without discrepancy and necessitates effective comprehensive multidisciplinary management [4].

The severity of back pain can be assessed by using various measures [11], and its effect on patients’ QOL is measured using valid tools [12,13]. Of these tools, the modified Oswestry low back pain disability index, World Health Organization quality of life scale (WHOQOL-BREF), SF36 questionnaire, and Quebec’s scale have been recommended for assessing the effect of physical and medical therapy in managing cases of cLBP [14].

This study aims to examine the prevalence of cLBP among adults seeking medical services in primary healthcare settings in Makkah City to evaluate the association of cLBP on HRQOL and to determine the predictors of subsequent disability among these patients.

## Materials and methods

A cross-sectional analytical study was performed to estimate the cLBP prevalence, its effect on HRQOL, and predictors of subsequent disability among adults attending primary care settings in Makkah City. The sample comprised patients who had attended outpatient primary healthcare centers seeking medical services from January 2023 to April 2023.

The study was conducted in Makkah, a city of great religious significance in Islam and a prominent location in the western region of Saudi Arabia. Consequently, there exists a significant variation in the social, cultural, and educational backgrounds of the population. The city has a population of over two million individuals, who are distributed among 60 distinct districts. The placement of primary healthcare centers (PHCCs) is determined by residential communities. The health authorities have implemented a division of Makkah City into four distinct areas, namely, north, east, west, and south. Sampling and randomization procedures were conducted in accordance with the distribution of PHCCs. A total of 10 accredited PHCCs from all four districts were first selected in a random manner from the list of PHCCs located in the specified area. Subsequently, patients were selected equally from each center by using systemic random samples from the selected PHC centers until the required sample size was reached.

The calculated sample size was n = 765; after adding 20% dropout, the final sample size was 918 subjects using the common formula for cross-sectional studies for 46.4% prevalence of low back pain, with a 5% margin of error, and 95% confidence interval (CI) and two for the design effect [15]. Patients who complained of LBP for more than 12 weeks were diagnosed with cLBP and were referred to a rheumatology outpatient clinic to be included for further assessment.

Inclusion criteria for further assessment

We included all adults (18 years and older) complaining of activity-related LBP for more than 12 weeks due to intrinsic spine causes (LBP due to muscular, intervertebral disc, facet, vertebral pathologies, and the surrounding soft tissues), with or without radicular symptoms (pain radiating to the knee or feet, numbness and/or muscle weakness of lower limbs, or altered responses of reflexes).

Exclusion criteria from further assessment

Further assessment excluded patients who were pregnant, patients with intra-pelvic pathology that is thought to be the cause of referred pain to their lower back, patients with osteoporosis documented by bone mineral density (BMD) and dual-energy X-ray absorptiometry (Dexa scan), patients with inflammatory back pain as ankylosing spondylitis or other seronegative spondyloarthropathies, and patients with any other autoimmune diseases that may affect the spine as confirmed by blood work and imaging studies. In addition, patients with a current or past history of tumors, previous surgery, or significant trauma to the spine were excluded.

Clinical evaluation

All selected patients were subjected to detailed history taking, including the onset, course, duration, and nature of the pain (mechanical or inflammatory); other associating symptoms denoting systemic affection; history of osteoporosis; other autoimmune diseases; and previous trauma of the spine. Neurological history taking (motor weakness or sensory affection of both lower limbs or bowel and/or bladder incontinence) was also performed.

In addition, patients were subjected to a thorough physical examination, focusing on the examination of the spine (inspection of curvatures and the covering skin for any abnormalities, suggesting underlying inflammation, palpation for any tenderness denoting inflammation, fractures, or metastasis). We examined the spine’s range of motion (measured by a bubble goniometer) and forward flexion (measured using Schober and modified Schober tests), side-bending, and rotation, in addition to carrying out the occiput-to-wall distance test to exclude inflammatory back pain. Further, patients were subjected to a full neurological examination of both lower limbs (motor, sensory, and reflexes). This examination included straight-leg raising and crossed-straight-leg raising tests (SLRT), to check for sciatica and lower lumbar nerve root irritation, and femoral stretch tests (FST), to check for upper lumber nerve root compression. Finally, a detailed abdominal examination was also conducted to exclude any referred pain from internal organs.

The severity of back pain was assessed using the visual analog scale of pain (VAS), a horizontal, straight, fixed-length indicator line (100 mm). The left end of the scale, 0, represents no pain, while the right end, 10, represents the worst suffering experienced. Patients make an indentation on the indicator line at the location that best captures their impression of the pain. Then, the VAS score is calculated using a ruler and measuring the distance in millimeters (mm) between the left end of the line and the patient’s marked point. The measured distance yields values between 0 and 100, with a higher number denoting more intense discomfort. The range from 0 to 4 mm denotes no pain; 5-44 mm, mild pain; 45-74 mm, moderate pain; and 75-100 mm, severe pain.

Questionnaires used in the study

A semi-structured questionnaire was designed to collect the following data from the patients:

· Socio-demographic data: age, gender, education, occupation, residence, marital status, and economic level

· Health data: past and health-seeking behavior risk factors and family history

· Clinical data on back pain: onset, course, duration, previous management, complications, neurological manifestations, and data on current radiological evaluation

The WHOQOL-BREF questionnaire, which is derived from WHOQOL-100 and abbreviated and validated, was used to evaluate back pain’s impact on HRQOL. This tool includes 26 questions to evaluate overall QOL and its specific domains (physical, psychological, social relations, and environment). The QOL tool is scored from zero to 100. The greater the score in each domain, the better the QOL. Zero is considered the least desired QOL score, while 100 is the most desired.

The modified Oswestry low back pain disability index, which has been widely used to assess functional status in LBP, was used to assess physical disability. The cLBP disability scores were classified as follows: minimal (0-20%), moderate (21-40%), severe (41-60), crippled (61-80%), and bedridden (81-100%).

Radiological evaluation

Plain radiographs of the lumbosacral spine (anteroposterior and lateral views) were taken for all the patients to search for any pathological causes of their symptoms. A lumbosacral spine MRI was also carried out to detect any pathoanatomical lesions not apparent by plain X-ray or to confirm the findings of conventional radiography when they did not match the clinical findings of the patient. All the radio­graphic images were analyzed by a radiologist who was completely blinded to the medical history and physical findings of the patients.

Laboratory evaluation

Acute phase reactants (ESR and CRP) were performed for all the subjects to exclude the presence of any inflammatory element causing their cLBP.

Compliance with ethical standards

Ethical approval was sought from Umm-Al-Qura University (no. HAPO-02-K-012-2022-11-1221). An informed consent form was signed (or fingerprinted) by each participant before enrolment in our study and collecting any data.

Statistical analysis

Data analysis was carried out using RStudio (R version 4.2.2). Frequencies and percentages were used to express categorical data. Tests of normality were applied to the scores of QOL, VAS, and ODI scales, indicating the non-normal distribution of the scores (p < 0.0001). Therefore, median and IQR were used to present continuous data. Additionally, we used non-parametric inferential tests to explore the statistical differences between different patient groups in terms of the QOL and disability scores. These included a Wilcoxon rank sum test for variables with two categories and a Kruskal-Wallis rank sum test for variables with more than two categories. The significantly associated variables from the inferential analysis were subsequently used in a multivariable generalized linear model to assess the predictors of disability using the disability score as a dependent variable. Results were expressed as beta coefficients and 95%CIs. A p-value of < 0.05 indicated statistical significance.

## Results

Prevalence of cLBP

**Figure 1:**
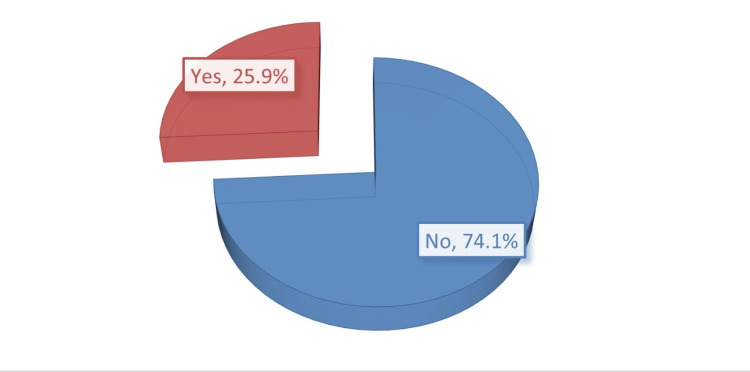
Prevalence of chronic low back pain among patients under study.

The prevalence of cLBP was calculated in this study; the numerator was the 238 patients with cLBP, and the denominator was the 918 adults who attended PHC settings seeking any medical service. Thus, 25.9% of patients were found to have chronic LBP (Figure 1). Data from 238 patients were collected in the current study. However, we excluded the records of 28 patients who potentially had an inflammatory cause of LBP, as indicated by the abnormal ESR (n = 26) and CRP results (n = 2). Therefore, we analyzed the data from 210 patients. Regarding sociodemographic and back pain-related characteristics, more than half of the patients were females (61.9%), aged 41-55 years (51.9%), and were not working (59.0%). Pain emerged gradually among 84.6% of the sample, and it aggravated with flexion among 53.3%. Constitutional and specific symptoms were prevalent among 8.6% and 21.0% of patients, respectively (Table 1).

**Table 1:** Sociodemographic and back pain-related characteristics (n = 210). *The variable has two missing values.

Parameter	Category	N (%)
Age (years)	≤ 40	36 (17.1%)
	41-55	109 (51.9%)
	> 55	65 (31.0%)
Gender	Female	130 (61.9%)
	Male	80 (38.1%)
Occupation	Not working	124 (59.0%)
	Working	86 (41.0%)
History of spine trauma	Absent	160 (76.2%)
	Present	50 (23.8%)
Onset of back pain*	Sudden	32 (15.4%)
	Gradual	176 (84.6%)
Aggravation of back pain	Flexion	112 (53.3%)
	Extension	62 (29.5%)
	Standing	24 (11.4%)
	Walking	12 (5.7%)
Constitutional symptoms	Absent	192 (91.4%)
	Present	18 (8.6%)
Associated symptoms	Absent	166 (79.0%)
	Present	44 (21.0%)

Outcomes of the diagnostic evaluations

On the clinical examination, all the patients had tenderness, and 88.6% of them had a limited range of motion of the spine. Scoliosis was reported among 18.1% of them. Positive SLRT and femoral stretch tests (FSTs) were indicated among 58.1% and 28.6%, respectively. Specific back pain, defined as pain associated with having an abnormal X-ray or MRI spine finding, was prevalent among 97.1% of patients under study (Table 2).

**Table 2:** Outcomes of the diagnostic studies for patients under study. *Specific pain was defined as pain associated with having abnormal X-ray or MRI findings.

Parameter	Category	N (%)
Clinical examination	Pain	206 (98.1%)
	Conscious	210 (100.0%)
	Abnormal vitals	0 (0.0%)
	Scoliosis	38 (18.1%)
	Tenderness	210 (100.0%)
	Limited ROM of the spine	186 (88.6%)
Neurological examination	Positive SLRT	122 (58.1%)
	Positive Lasgue manauver	10 (4.8%)
	Positive femoral stretch test	60 (28.6%)
	Positive rectal tones	6 (2.9%)
Radiological examination	Abnormal X-ray	200 (95.2%)
	Abnormal MRI of the lumbosacral spine	122 (58.1%)
	Abnormal CT	54 (25.7%)
Specific pain*	Yes	204 (97.1%)

Description of the used questionnaires

**Figure 2:**
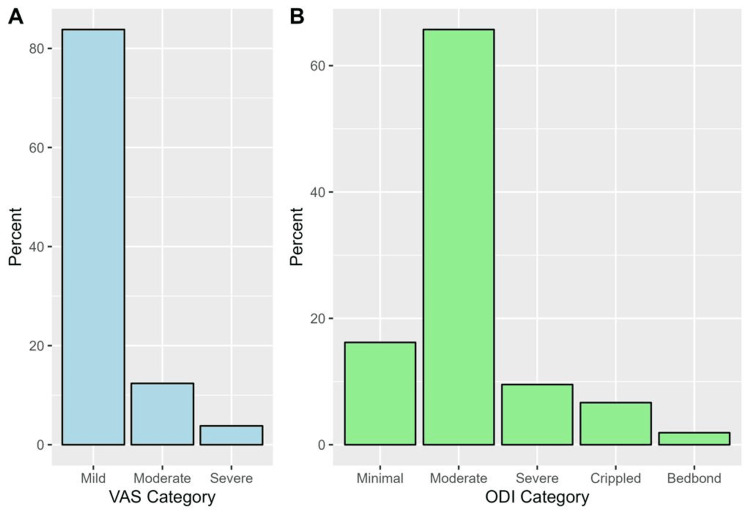
Proportions of different categories of the VAS scale (A) and the ODI questionnaire (B).

The numerical scores of the QOT, VAS scale, and ODI questionnaire are described in Table 3. The median (IQR) QOL score was 70 (60-80). Based on the VAS scale, the majority of patients with back pain had mild pain (83.8%, Figure 2A). Minimal and moderate disability were prevalent among 16.2% and 65.7% of patients, respectively (Figure 2B).

**Table 3:** Description of the scores of questionnaires used in the current study. ODI: Oswestry disability index; VAS: Visual analogue scale

Domain	Median (IQR)	Mean ± SD	Min - Max
Quality-of-life score	70 (60-80)	66.3 ± 14.6	20-90
VAS score	33 (23-39)	35.1 ± 16.9	13-87
ODI score	29 (22-39)	33.2 ± 15.6	13-87

Factors associated with the QOL

The QOL score differed significantly based on patients' ages (p = 0.008), limited ROM of the spine (p = 0.047), abnormal MRI findings (p = 0.033), and PVRV results (p = 0.003) (Table 4).

**Table 4:** Differences in the scores of quality of life and disability among patients under study (n = 210). QOL: quality of life; ODI: Oswestry low back pain disability index; IQR: interquartile ranges; SLRT: straight leg raising tests; PVRV: post-void residual volume

Parameter	Category	QOL score			ODI score		
		Median	(IQR)	p-value	Median	(IQR)	p-value
Age (years)	≤ 40	70.0	(60.0, 80.0)	0.008	34.0	(26.0, 37.0)	0.073
	41-55	70.0	(60.0, 80.0)		27.0	(22.0, 37.0)	
	> 55	70.0	(40.0, 70.0)		33.0	(23.0, 57.0)	
Gender	Female	70.0	(60.0, 80.0)	0.086	29.0	(22.0, 39.0)	0.515
	Male	70.0	(60.0, 70.0)		29.5	(24.8, 38.5)	
Occupation	Not working	70.0	(60.0, 80.0)	0.077	30.5	(22.0, 40.0)	0.486
	Working	70.0	(60.0, 80.0)		29.0	(22.2, 36.8)	
Onset of back pain	Sudden	65.0	(60.0, 72.5)	0.198	36.0	(25.5, 41.5)	0.017
	Gradual	70.0	(60.0, 80.0)		29.0	(22.0, 37.0)	
Aggravation of back pain	Flexion	70.0	(60.0, 72.5)	0.074	30.0	(22.8, 39.0)	0.245
	Extension	70.0	(60.0, 80.0)		32.0	(23.2, 37.0)	
	Standing	60.0	(55.0, 72.5)		32.5	(20.8, 61.2)	
	Walking	70.0	(70.0, 80.0)		26.0	(18.0, 30.0)	
Constitutional symptoms	Absent	70.0	(60.0, 80.0)	0.342	30.5	(22.0, 39.0)	0.729
	Present	70.0	(60.0, 80.0)		28.0	(25.0, 37.0)	
Associated symptoms	Absent	70.0	(60.0, 80.0)	0.119	29.0	(22.0, 40.0)	0.751
	Present	70.0	(60.0, 80.0)		30.5	(25.0, 37.0)	
History of spine trauma	Absent	70.0	(60.0, 80.0)	0.638	29.0	(22.0, 37.5)	0.090
	Present	70.0	(60.0, 70.0)		33.0	(24.0, 40.0)	
Pain	No	70.0	(60.0, 80.0)	0.733	26.0	(13.0, 39.0)	0.399
	Yes	70.0	(60.0, 80.0)		29.0	(22.2, 38.8)	
Scoliosis	No	70.0	(60.0, 80.0)	0.103	29.0	(22.0, 39.0)	0.646
	Yes	70.0	(60.0, 70.0)		30.0	(21.5, 40.0)	
Limited ROM of the spine	No	70.0	(67.5, 80.0)	0.047	25.5	(20.2, 29.2)	0.010
	Yes	70.0	(60.0, 80.0)		33.0	(23.0, 40.0)	
Positive SLRT	No	70.0	(60.0, 72.5)	0.422	29.0	(20.5, 36.2)	0.051
	Yes	70.0	(60.0, 80.0)		32.0	(23.0, 40.0)	
Positive Lasgue manauver	No	70.0	(60.0, 80.0)	0.228	29.5	(22.8, 39.2)	0.217
	Yes	70.0	(70.0, 80.0)		23.0	(22.0, 32.0)	
Positive Femoral Stretch Test	No	70.0	(60.0, 80.0)	0.570	27.0	(21.2, 36.8)	<0.001
	Yes	70.0	(60.0, 80.0)		35.5	(25.0, 42.0)	
Positive rectal tones	No	70.0	(60.0, 80.0)	0.694	29.0	(22.0, 39.0)	0.626
	Yes	70.0	(62.5, 77.5)		32.0	(26.8, 38.0)	
Abnormal X-ray	No	80.0	(70.0, 80.0)	0.024	20.0	(18.0, 26.0)	0.025
	Yes	70.0	(60.0, 80.0)		30.5	(23.0, 39.0)	
Abnormal MRI lumbosacral spine	No	70.0	(60.0, 80.0)	0.033	26.0	(18.0, 34.0)	<0.001
	Yes	70.0	(60.0, 70.0)		34.0	(24.0, 40.0)	
Abnormal CT	No	70.0	(60.0, 80.0)	0.388	28.5	(22.0, 40.0)	0.383
	Yes	70.0	(60.0, 77.5)		33.0	(25.5, 36.0)	
PVRV	Absent	70.0	(60.0, 80.0)	0.003	29.0	(22.0, 37.0)	<0.001
	Present	60.0	(30.0, 70.0)		40.0	(27.0, 65.0)	
Specific pain	No	80.0	(65.0, 87.5)	0.082	18.0	(16.5, 24.0)	0.007
	Yes	70.0	(60.0, 80.0)		30.5	(23.0, 39.0)	

Factors associated with disability

**Figure 3:**
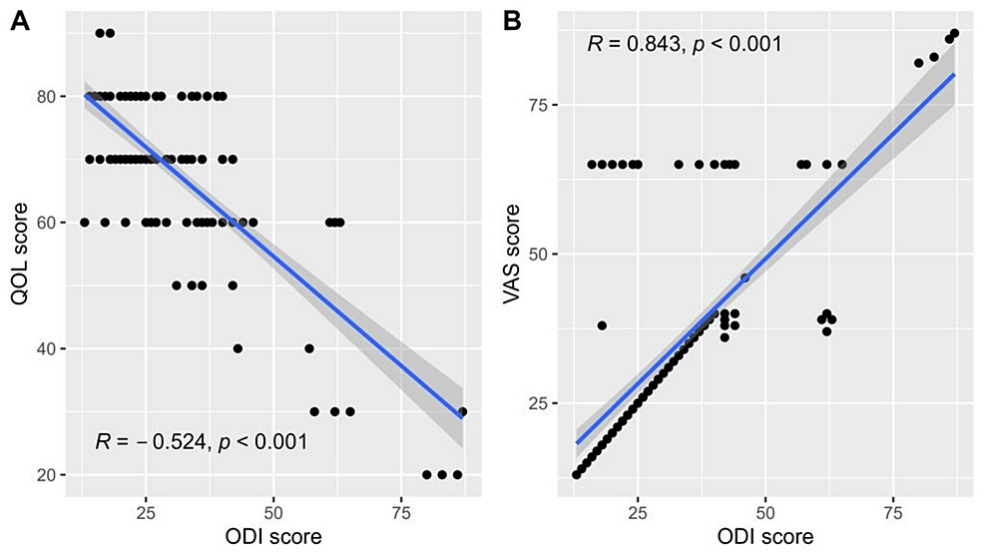
Scatterplots depicting the correlations between the ODI score and the quality-of-life score (A) and the VAS score (B).

Regarding the disability score, the score differed significantly based on the onset of pain (p = 0.017), spine range of motion (p = 0.010), the outcomes of FST (p < 0.001), having abnormal X-ray (p = 0.025), having abnormal MRI lumbosacral spine (p < 0.001), PVRV (p < 0.001) and having specific back pain, defined as pain associated with having an abnormal X-ray or MRI spine finding (p = 0.07; Table 4). Additionally, disability scores correlated negatively with the QOL score (p < 0.001, Figure 3A) and positively with the VAS score (p < 0.001, Figure 3B).

Predictors of disability

In the multivariable regression analysis, we planned to use the significantly associated variables from the inferential analysis as independent variables in the model. These included the following variables: the onset of pain, spine range of motion, a positive FST, having an abnormal X-ray result, having abnormal MRI lumbosacral spine, PVRV, and having specific pain. However, we have to eliminate the specific pain variable since the pain has been associated with having abnormal lumbosacral spine images on X-ray or MRI in order to prevent misinterpretation and the expected multicollinearity, and we decided to keep the variable of abnormal X-ray and MRI. The analysis was adjusted since the significantly associated variables were selected and incorporated in a multivariable regression model using the Enter method. Results of the regression analysis demonstrated that disability scores were independently predicted by lower QOL scores (beta = -0.39, 95%CI = -0.50 to -0.29, p < 0.001) and higher VAS scores (beta = 0.46, 95%CI = 0.37-0.54, p < 0.001). Furthermore, disability was independently associated with having PVRV (beta = 5.81, 95%CI = 1.87-9.74, p = 0.004), as well as having abnormal X-ray findings (beta = 7.10, 95%CI = 1.77-12.4, p = 0.010, Table 5).

**Table 5:** Result of the predictors of disability among patients under study. CI: confidence interval; Ref: reference group; QOL: quality of life; VAS: visual analog scale; ROM: range of motion; PVRV: post-void residual volume; MRI: magnetic resonance imaging

Characteristic	Beta	95% CI	p-value
QOL score	-0.39	-0.50, -0.29	<0.001
VAS	0.46	0.37, 0.54	<0.001
Onset of back pain			
Sudden	Ref	Ref	
Gradual	-1.79	-5.15, 1.58	0.299
Limited ROM of the spine			
No	Ref	Ref	
Yes	-2.60	-6.23, 1.03	0.162
Positive Femoral Stretch Test			
No	Ref	Ref	
Yes	2.36	-0.28, 5.01	0.081
PVRV			
Absent	Ref	Ref	
Present	5.81	1.87, 9.74	0.004
Abnormal X-ray			
No	Ref	Ref	
Yes	7.10	1.77, 12.4	0.010
Abnormal MRI of lumbosacral spine			
No	Ref	Ref	
Yes	1.44	-1.13, 4.00	0.273

## Discussion

cLBP is a prevalent multifactorial health problem. It affects QOL through pain, functional disability, and psychosocial distress. This significant impact on QOL results in significant costs to society [16,17]. In this work, we estimated the prevalence of cLBP adults who attended primary healthcare centers seeking any medical service and estimated its impact on their QOL. Our results revealed a prevalence for cLBP of about 25.9%, which was consistent with previous estimates suggesting a prevalence of 23% [18]. Most studies concluded similar findings as the prevalence of cLBP varies according to the age group from 14% among 18-25 years old and 25%-45% among relatively older age groups [19]. In the current study, 82.9% of patients with cLBP were above 40 years, and 61.9% were females. Our results also were consistent with several previous studies that revealed the more frequent prevalence of cLBP in women and older age groups [20-22]. This may be explained by the sedentary lifestyle as most study subjects were not working and higher rates of obesity were seen in females and elders.

In our study, we found that only 2.9% of the patients had no definite cause for their pain (apparent on either plain radiography or MRI); in contrast, another study found that up to 90% of patients with cLBP may have no definite cause for this pain [23]. This discrepancy between our results and the results of the previous study might be due to considering lumbar spondylolysis or spondylolisthesis and spina bifida occulta as non-specific cLBP because a large proportion of patients with such anatomic abnormalities are asymptomatic [2,24].

According to previously reported results of the Oswestry survey, subjects with cLBP have a significantly higher disability score and lower QOL score [11,21,24]. The mean Oswestry low back pain disability index score in our study was 33.2±15.6, with the majority of the patients (65.5%) having a moderate disability. This can be explained by the fact that the correlation of pain intensity as detected by VAS and the disability score may lead to fear of movement and subsequent disability. Other studies revealed that aggravation of pain intensity increases the physical disability of the patient, starting a worsening cycle of depression, pain perception, physical inactivity, and disability [11,25].

The median QOL score in this study was 70 (60-80), which was higher than the results in a Cameron study, which revealed that the median QOL score was 50 (IQR: 25). This difference might be due to the elder study subjects in the previous study as the mean age was 52 years. We found that the median QOL was significantly related to age, limited movement, and abnormal radiologic findings. Similar associations were reported in another study, which showed that age, education, smoking, and abnormal imaging were associated with poor QOL [26].

Upcoming disability, estimated by multiple linear regression analysis, was positively predicted by the VAS and abnormal X-ray findings and negatively predicted by the QOL score. Disability scores were independently predicted by lower QOL scores and higher VAS scores. Furthermore, disability was independently associated with having PVRV. These results could help in the early detection and avoidance of increasing pain and disability in patients with cLBP. Similarly, a fear of movement, pain severity, anxiety, and depression are consistently stated to predict disability. This highlights the importance of addressing these factors in the management of cLBP [24,27].

Limitations of the study

The study was a cross-sectional analysis that could not explain a causal association, and a longitudinal study design will be needed to follow up on the cases and their progression to confirm the prediction of disability. In addition, it was conducted in primary care settings and in Makkah City only, so it could not be generalized to the general population.

## Conclusions

cLBP was common in adults attending primary care settings in Makkah City, with a higher frequency in women than men. It was associated with moderate disability and lower HRQOL. Disability scores were correlated negatively with the QOL scores and positively with the VAS scores. The findings highlight the significant impact of cLBP on disability, as well as HRQOL.
